# Supplementary material for: Changes in Soil Phosphorus Availability and Microbial Community Structures in Rhizospheres of Oilseed Rapes Induced by Intercropping with White Lupins
Source: Microorganisms. 2023 Jan 28;11(2):326. doi: 10.3390/microorganisms11020326 (PMC9959241; doi:10.3390/microorganisms11020326)
Supplement: Supplementary file 1 [file microorganisms-11-00326-s001.zip › microorganisms-2134425-supplementary.pdf]

Table S1. The metabolites Lon (M/Z) and positive/negative ionization mode of differential root exudates in rhizospheres of oilseed rapes between RR and RL systems.

Metabolite	Lon (M/Z)	Positive/negative
Organic acids and derivatives		
N-Undecanoylglycine	226.18	ESI+
Citric acid	215.02	ESI+
Creatine	132.08	ESI+
ADMA	203.15	ESI+
Tryptophyl-cysteine	325.13	ESI+
Isoleucylproline	211.14	ESI+
2-Amino-4-[(2-hydroxy-1-oxopropyl)amino] butanoic acid	155.08	ESI+
L-trans-5-Hydroxy-2-piperidinecarboxylic acid	128.07	ESI+
Lysyl-valine	532.38	ESI+
Leucyl-threonine	215.14	ESI+
Organic oxygen compounds		
5-Hydroxy-6-methoxyindole glucuronide	340.11	ESI+
Aminoacetone	74.06	ESI+
Organic nitrogen compounds		
L-Carnitine	162.11	ESI+
Memantine	180.17	ESI+
Tromethamine	122.08	ESI+
Organoheterocyclic compounds		
Cis-quinceoxepane	198.19	ESI+
9-Azabicyclo[3.3.1] nonan-3-one	157.13	ESI+
5-Oxo-2(5H)-isoxazolepropanenitrile	177.01	ESI+
Phenylpropanoids and polyketides		
2-Pentyl-3-phenyl-2-propenal	443.23	ESI+
Nucleosides, nucleotides, and analogs		
Lamivudine sulfoxide	266.02	ESI-
7-Methylinosine	266.10	ESI+
Lipids and lipid-like molecules		
Aucubin	311.16	ESI+
Etonogestrel	388.22	ESI+
4,5-Dihydrovomifoliol	209.15	ESI+
(2S,4R)-p-Mentha-1(7),8-dien-2-ol	327.23	ESI+
Dihydrozeatin-O-glucoside	348.17	ESI+
Benzenoids		
Benzaldehyde	107.05	ESI+
4-Hydroxy-5-phenyltetrahydro-1,3-oxazin-2-one	176.07	ESI+
2-Dodecylbenzenesulfonic acid	325.18	ESI-
Other		
NORETHINDRONE ACETATE	339.20	ESI-
3-methyl-2-Quinoxalinone	161.07	ESI+
13-Hydroxylupanine	265.19	ESI+
Allothreonine	120.07	ESI+
Eicosanoyl-EA	356.35	ESI+
5-Hydroxyindol-2-carboxylic acid	178.05	ESI+

3,5-dihydroxy-2-(3-hydroxyphenyl)-8,8-dimethyl-4H,8H-pyrano[2,3-f] chromen-4-one	353.10	ESI+
3,4,5-trihydroxy-6-[(2-phenylacetyl)oxy] oxane-2-carboxylic acid	277.07	ESI+
2-Deoxy-2-dimethylamino-alpha-D-Glucose	208.12	ESI+
2-AI	134.10	ESI+
3-(5,7-dihydroxy-2,2-dimethyl-3,4-dihydro-2H-1-benzopyran-6-yl)-7-hydroxy-4H-chromen-4-one	319.10	ESI+
8-(1,2-dihydroxypropan-2-yl)-9-hydroxy-2H,8H,9H-furo[2,3-h] chromen-2-one	296.11	ESI+
Phenylenediamine	109.08	ESI+
